# EGCG regulates the cross-talk between JWA and topoisomerase IIα in non-small-cell lung cancer (NSCLC) cells

**DOI:** 10.1038/srep11009

**Published:** 2015-06-05

**Authors:** Yuan Li, Xin Shen, Xueming Wang, Aiping Li, Pengqi Wang, Pan Jiang, Jianwei Zhou, Qing Feng

**Affiliations:** 1 Department of Nutrition and Food Hygiene, School of Public Health, Nanjing Medical University, Nanjing, Jiangsu, 211166, China; 2 Rizhao Centers For Disease Control and Prevention, Rizhao, Shandong, 276826, China; 3 Department of Molecular Cell Biology and Toxicology, Cancer Center, School of Public Health, Nanjing Medical University, Nanjing, Jiangsu, 211166, China

## Abstract

(-)-epigallocatechin-3-gallate (EGCG) is a well-known cancer chemopreventive agent. The potential mechanisms include regulation of multiple molecules. Carcinogenesis in lung cancer is related to the imbalance of tumor suppressor and oncogene. JWA is a structurally novel microtubule-binding protein and is a potential tumor suppressor. DNA topoisomerase IIα is a nuclear enzyme that governs DNA topology and is usually highly expressed in many types of cancer. It serves as a target of anticancer drugs. In the current study, the regulation of JWA and topoisomerase IIα by EGCG, and thereafter the mutual interaction between them was investigated. The results revealed that EGCG up-regulated JWA while decreased topoisomerase IIα expression in both human non-small cell lung cancer (NSCLC) cells and an NSCLC xenograft mice model. There was a negative correlation between JWA and topoisomerase IIα in NSCLC as well as in human NSCLC tissue specimens. Topoisomerase IIα overexpression reduced JWA at the translational level. Meanwhile, JWA-induced topoisomerase IIα degradation was regulated both in the transcriptional and post-translational level. Interestingly, JWA and topoisomerase IIα regulated each other in the cells arrested in G2/M. Furthermore, JWA and topoisomerase IIα synergistically affected NCI-H460 cells invasion. These results may serve a novel mechanism for cancer prevention.

Lung cancer is a leading cause of cancer death. Non-small-cell lung cancer (NSCLC) represents approximately 85% of lung cancer cases, with a world-wide annual incidence of approximately 1.3 million^1^. Advances in the understanding of specific molecular abnormalities can provide new strategies for personalized lung cancer treatment including gene amplifications (e.g., MET, FGFR1), mutations (e.g., EGFR, p53) and fusions (e.g., EML4-ALK)^2^. Chemoprevention is a promising strategies interfering carcinogenesis. EGCG, a major active polyphenol, has captured much attention as a potential cancer chemopreventive agent^3^,^4^. Previous studies have revealed the possible molecular mechanisms of EGCG to control lung cancer insurgence^5^,^6^,^7^.

JWA is known as adenosine diphosphate-ribosylation-like factor 6 interacting protein 5 (ARL6ip5) in GenBank (AF070523). It was initially cloned from human tracheal bronchial epithelial cells after treatment with all-trans retinoic acid (ATRA). JWA encodes a structurally novel microtubule-associated protein, which regulates cancer cells differentiation and apoptosis induced by multiple chemicals^8^,^9^. JWA responses to environmental stimulations including heat shock and H_2_O_2_-induced oxidative stress^10^,^11^. It has been reported that JWA may serve as a repair protein by regulating base excision repair (BER) protein XRCC1^12^. On the other hand, JWA is known as a novel tumor suppressor which regulates tumor angiogenesis by suppressing matrix metalloprotein (MMP) and inhibiting cell invasion via focal adhesion kinase (FAK/PTK2)^13^. Further investigations indicate that JWA can work as a cooperator with p53, MDM2 or XRCC1 to improve predictive potency in gastric cancer^14^,^15^,^16^. Moreover, JWA sensitizes p-glycoprotein-mediated drug resistance to anticancer drug etoposide (topoisomerase II inhibitor)^17^.

DNA topoisomerases are ubiquitous nuclear enzymes that govern DNA topology and fundamental DNA processes involved in DNA replication, transcription, chromosome condensation and recombination^18^. There are two main types of the enzyme, catalyzing transient breaks in one (type I) or both (type II) strands of DNA. In the topoisomerase II family, topoisomerase IIα and topoisomerase IIβ are homologous sharing extensive amino acid sequence identity (~70%). However, the two isoforms have distinct patterns of expression^19^. Topoisomerase IIα is cell cycle-dependent and primarily expresses in rapidly proliferating cells. High levels of this isoform are found in many types of cancer, therefore it is a cancer target in clinical application^20^,^21^. The chemotherapeutic properties are attributed primarily to topoisomerase IIα^22^. Although topoisomerase II-mediated DNA cleavage has been recognized as an effective molecular target for many antitumor drugs^23^, frequently experienced occurrence of serious side effects of these molecules during therapy have been reported^24^.

It is reported that EGCG is redox-dependent topoisomerase II poison^25^,^26^. It enhances DNA cleavage and affects topoisomerase activity mediated by both enzyme isoforms^27^,^28^. In the present study, we find EGCG could also suppress topoisomerase IIα expression. Interestingly, it also up-regulated JWA. The underlying mechanism of the relationship between JWA and topoisomerase IIα was investigated. Whether EGCG participated in the regulation of JWA and topoisomerase IIα in NSCLC cells was explored. Furthermore, the combination of JWA and topoisomerase IIα might serve as a novel candidate prognostic biomarker for NSCLC.

## Results

### EGCG induced expression of JWA in NSCLC cells

**Figure 1:**
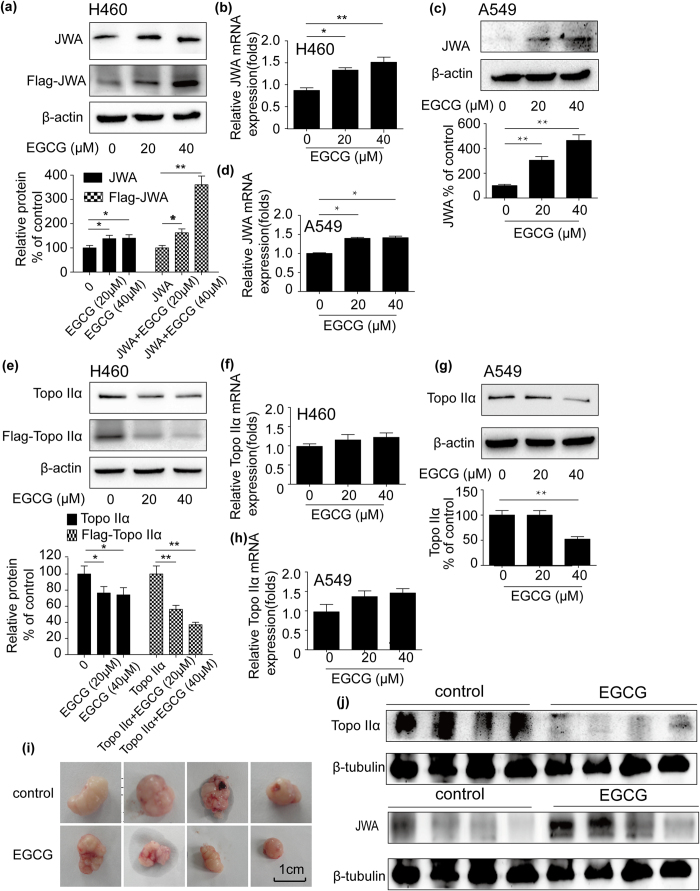
EGCG regulated the expression of JWA and topoisomerase IIα (topo IIα) *in vitro* (NCI-H460 and A549 cells) and *in vivo*. (**a**) Cells transfected with Flag-JWA (4 μg) or without transfection were incubated with EGCG (20–40 μM) for 24 h. JWA protein expression was assessed by Western blot analysis. β-actin expression served as a loading control. (**b**) NCI-H460 cells were treated with EGCG (20–40 μM) for 24 h and total cellular RNA was extracted. mRNA level of JWA was detected by real-time PCR. GAPDH was used as an internal control. (**c**) Western blot analysis of the protein level of JWA in EGCG-treated A549 cells for 24 h. β-actin expression served as a loading control. (**d**) After A549 cells were incubated with EGCG (20–40 μM) for 24 h, total RNAs were prepared and real-time PCR was applied to measure the JWA mRNA level. GAPDH was used as an internal control. (**e**) NCI-H460 cells were transfected with or without Flag-topoisomerase IIα plasmid (4 μg) and then treated with EGCG (20–40 μM) for 24 h. Protein from cell was subjected to western blot analysis. β-actin expression was served as a loading control. (**f**) Total RNAs from NCI-H460 cells incubated with EGCG (20–40 μM) for 24 h were extracted and subjected to real-time PCR using primer for topoisomerase IIα. GAPDH was used as an internal control. (**g**) A549 cells were treated with EGCG (20–40 μM) for 24 h and then protein from cell lysate was subjected to western blot analysis. β-actin expression was served as a loading control. (**h**) A549 cells were incubated in the absence or presence of EGCG (20–40 μM) for 24 h. Then cells were lysed for the detection expression of topoisomerase IIα mRNA by real-time PCR. GAPDH was used as an internal control. The A549 xenograft nude mice model was established and treated with EGCG or normal saline. (**i**) Tumor size was checked twice per week. (**j**) Protein obtained from tumor tissues was subjected to western blot. β-tubulin expression was served as a loading control. Error bars represent the mean ± SD of triplicate experiments. Statistical differences to the controls were shown as *p < 0.05, **p < 0.01.

Firstly, the effect of EGCG on JWA expression was investigated in NSCLC cell lines. Total RNA or protein from A549 and NCI-H460 cells treated with indicated concentration of EGCG was isolated respectively. Western blot analysis was used to detect endogenous and exogenous JWA protein level. As shown in Fig. 1a, EGCG up-regulated endogenous JWA protein level in NCI-H460 cells in a dose-dependent manner. When the same concentrations of EGCG were treated to the cells transfected with Flag-JWA plasmid, the exogenous JWA protein level, as tested by anti-Flag antibody, also increased. Then, real-time PCR was preformed to examine JWA mRNA expression. As shown in Fig. 1b, EGCG increased JWA messenger RNA (mRNA) level as well in NCI-H460 cells. Meanwhile, the same effect of EGCG on JWA expression was observed in A549 cells (Fig. 1c,d).

### EGCG suppressed topoisomerase IIα expression in NSCLC cells.

As EGCG is an inhibitor of topoisomerase II in leukaemia and hamster ovary cells^26^,^29^, whether EGCG shows inhibitory effect on topoisomerase II expression was investigated in NCI-H460 and A549 cells. Cells were treated with EGCG at the indicated concentrations for 24 h and followed by western blot assay. As shown in Fig. 1e,g, EGCG significantly inhibited protein expression of topoisomerase IIα in NCI-H460 and A549 cells. We further determined the effect of EGCG on expression of topoisomerase IIα mRNA by real-time PCR, The results revealed that topoisomerase IIα mRNA expression was closed to the level of control in both of the cells (Fig. 1f,h). The protein level of topoisomerase IIα was down-regulated by EGCG while mRNA level was not changed, which indicating post-transcription regulation of topoisomerase IIα by EGCG.

### EGCG increased the expression of JWA while down-regulated topoisomerase IIα levels in tumor-bearing nude mice

An A549 xenograft nude mice model was established to confirm the influence of EGCG on JWA and topoisomerase IIα expression *in vivo*. As shown in Fig. 1i, the tumor growth was repressed significantly by EGCG treatment for 30 days, compared to the control group. Treatment of EGCG altered the expression of JWA and topoisomerase IIα. Topoisomerase IIα protein expression was reduced in EGCG-treated mice compared with the control. On the contrary, JWA levels were increased after EGCG treatment (Fig. 1j). These results were consistent with the above experiments *in vitro*. These findings indicated that there was a negative correlation between JWA and topoisomerase IIα protein expression upon EGCG treatment.

### The expression of JWA and topoisomerase IIα in lung cancer versus adjacent normal tissues

**Figure 2:**
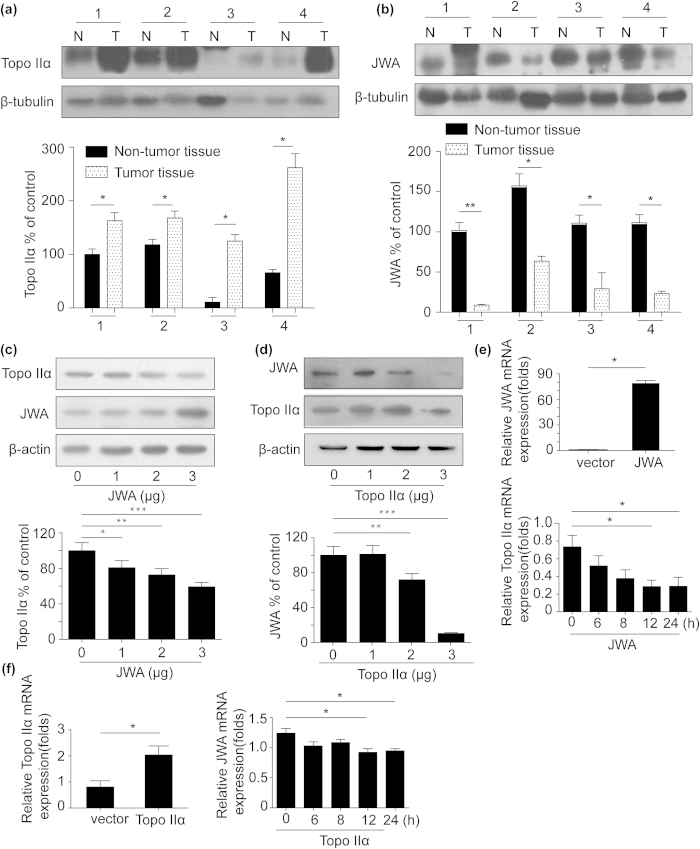
There was a negative correlation between JWA and topoisomerase IIα protein expression. (**a**) and (**b**) Expression of topoisomerase IIα was elevated and that of topoisomerase IIα was reduced in lung cancer tissues compared with those in matched normal tissues. β-Tubulin expression was served as a loading control. JWA and topoisomerase IIα expressions in cancer tissues (T) and paired non-cancerous normal tissues (N) of lung cancer patients were analyzed by Western blotting. The level of each protein was normalized against β-tubulin. NCI-H460 cells were transiently transfected with Flag-JWA or Flag- topoisomerase IIα plasmid (1–4 μg). Whole-cell extracts were prepared 24 h after transfection and the expression of target proteins and mRNAs was examined. As analyzed by western blot assay, topoisomerase IIα levels were decreased after transfection with JWA plasmid (**c**) Also, overexpression of topoisomerase IIα does dependently inhibited JWA protein expression (**d**) β-actin was used for the protein loading control. Total RNAs were isolated from NCI-H460 cells transfected with Flag-JWA or Flag- topoisomerase IIα plasmid (4 μg) and subjected to real-time PCR. JWA transfection suppressed topoisomerase IIα mRNA expression (**e**) The level of JWA mRNA was down-regulated in topoisomerase IIα overexpressed NCI-H460 cells (**f**) GAPDH was used as an internal control. Error bars represent the mean ± SD of triplicate experiments. Statistical differences to the controls were shown as *p < 0.05, **p < 0.01.

Four pairs of human NSCLC samples, including primary lung cancer tissues and matched non-cancerous tissues were selected to test JWA and topoisomerase IIα protein expression by western blot. Elevated expression of topoisomerase IIα and decreased JWA level were observed in lung cancer tissues compared with the paired normal tissues (Fig. 2a,b). The results showed a negative correlation between JWA and topoisomerase IIα expression in lung cancer versus non-cancer tissues.

### JWA and topoisomerase IIα reciprocally down-regulated in NSCLC cells

Since EGCG exhibited opposite effect on expression of JWA and topoisomerase IIα, we therefore postulated if there existed a converse-regulation between JWA and topoisomerase IIα. To verify this hypothesis, NCI-H460 cells were transfected with plasmids to overexpress JWA or topoisomerase IIα. The western blot analysis showed that JWA overexpression suppressed topoisomerase IIα levels in a dose-dependent manner (Fig. 2c). Interestingly, topoisomerase IIα dose-dependently inhibited JWA protein expression (Fig. 2d).

We further examined the topoisomerase IIα mRNA expression after transfection of JWA. Total RNAs were isolated from the cells and subjected to real-time PCR. As shown in Fig. 2e, transfection of JWA suppressed topoisomerase IIα mRNA expression. Also, the levels of JWA mRNA were decreased in NCI-H460 cells which topoisomerase IIα was overexpressed (Fig. 2f).

**Figure 3:**
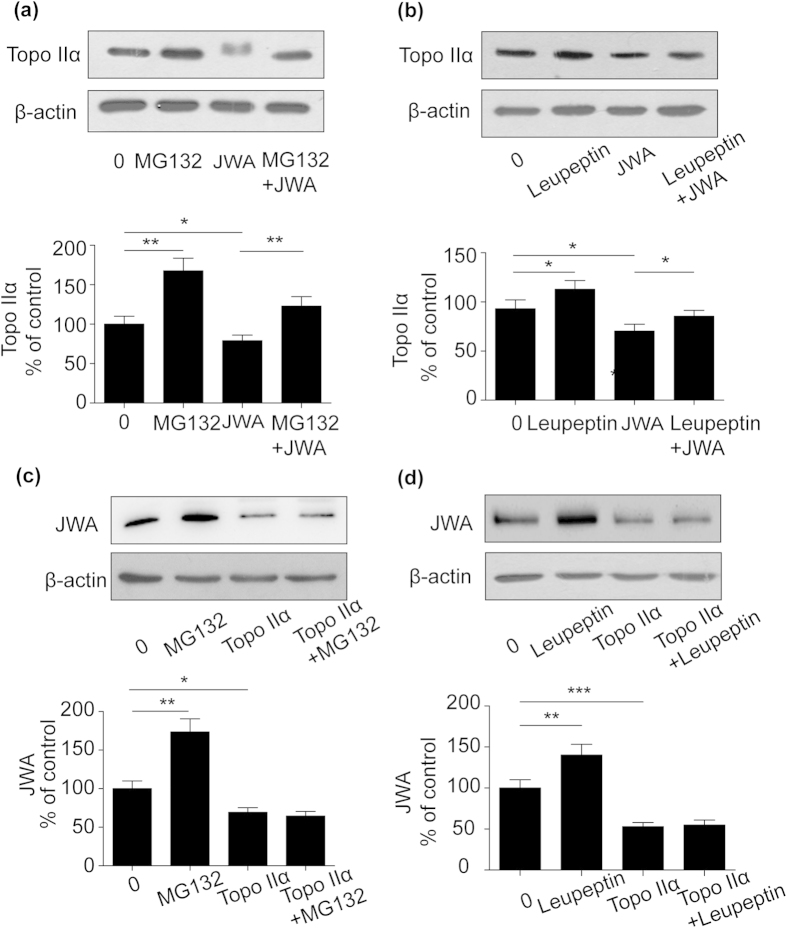
Different degradation pathway regulated the inverse interaction between topoisomerase IIα and JWA protein expression. (**a**) and (**b**) NCI-H460 cells were transiently transfected with Flag-JWA plasmid (2.5 μg) for 24 h, incubated with (+) or without (-) MG-132 (5 μM) for 8 h or leupeptin (5 μM) for 20 h. Western blotting was carried out to confirm the level of topoisomerase IIα. (**c**) and (d) NCI-H460 cells were transiently transfected with Flag- topoisomerase IIα plasmid (2.5 μg) for 24 h and co-treated with (+) or without (-) MG-132 (5 μM) for 8 h or leupeptin (5 μM) for 20 h. JWA protein was detected by anti-JWA antibody. β-actin expression served as a loading control. Error bars represent the mean ± SD of triplicate experiments. Statistical differences to the controls were shown as *p < 0.05, **p < 0.01.

Since the above results showed that JWA and topoisomerase IIα down-regulated each other at both mRNA and protein levels, we further confirmed whether the proteasome or lysosome pathway was responsible for the negative regulation between JWA and topoisomerase IIα. When NCI-H460 cells were treated with the proteasome inhibitor MG132, we observed that down-regulation of topoisomerase IIα in JWA overexpressed cells was blocked (Fig. 3a). Leupeptin, a lysosome inhibitor, was able to reverse the decreased topoisomerase IIα triggered by JWA (Fig. 3b). However, when the cells were transfected with topoisomerase IIα in the presence of leupeptin or MG-132, JWA level still decreased (Fig. 3c,d). These data revealed that both lysomal and proteasomal pathway were involved in JWA-mediated suppression of topoisomerase IIα. Nevertheless, topoisomerase IIα-induced JWA degradation might not occurred in the proteasome or lysosome.

### The amino acid sequence of JWA ranging from 90 to 188 played a crucial role in topoisomerase IIα degradation

**Figure 4:**
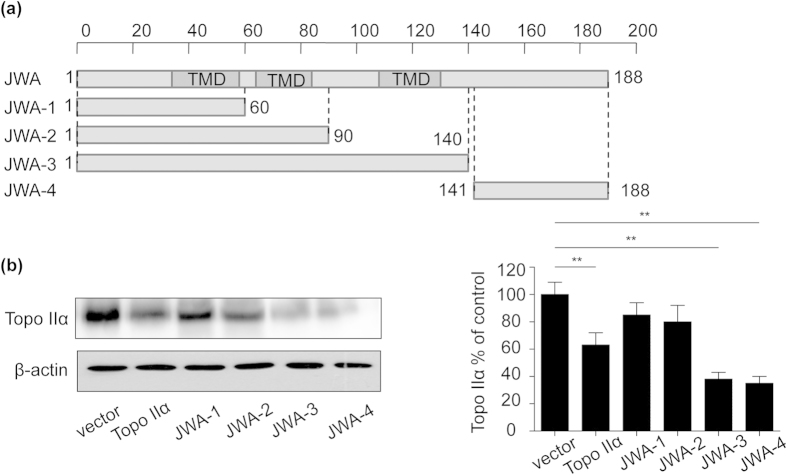
The deletion of 90–188 amino acid of JWA played a major function for topoisomerase IIα degradation. (**a**) Schematic showed the domains of full length and indicated fragments JWA protein. “Dark grey” represented the TMD of JWA. Amino acid sequences ranged from 1 to 60 for JWA-1, 1 to 90 for JWA-2, 1 to 140 for JWA-3 and 141–188 for JWA-4. (**b**) NCI-H460 cells were transiently transfected with full-length Flag-JWA plasmid and four different fragments of JWA plasmid respectively. Whole-cell extracts were prepared 24 h after transfection, and topoisomerase IIα expression was measured by western blotting. Error bars represent the mean ± SD of triplicate experiments. Statistical differences to the controls were shown as *p < 0.05, **p < 0.01.

To elucidate which fragments of JWA acted in topoisomerase IIα degradation, four fragments were constructed based on JWA functional domain, of which amino acid sequences were ranged from 1 to 60 (JWA-1), 1 to 90 (JWA-2), 1 to 140 (JWA-3) and 141–188 (JWA-4) (Fig. 4a). After transient transfection of these constructs’ plasmids into NCI-H460 cells, we examined the topoisomerase IIα protein expression by western blot analysis. It revealed significant down-regulation of topoisomerase IIα with JWA-3 (1–140) and JWA-4 (141–188) fragments compared with the control (Fig. 4b). However, no makeable difference was observed in cells transfected with JWA-1 (1–60) and JWA-2 (1–90). These results indicated that the region of JWA amino acid sequence between 90 and 188 played an important function for topoisomerase IIα degradation.

### The mutual regulation of JWA and topoisomerase IIα was occurred in G2/M

**Figure 5:**
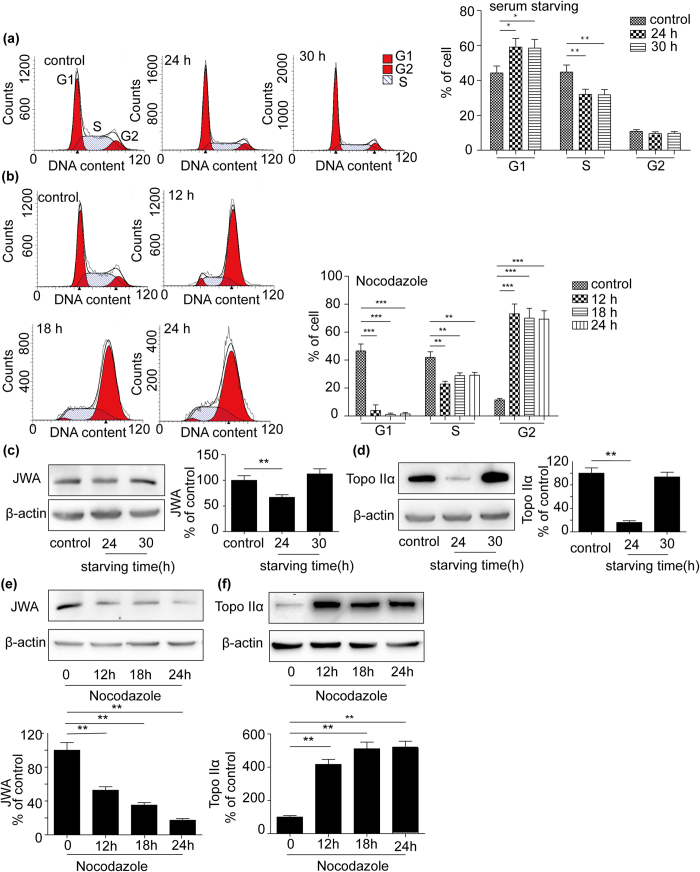
Cell cycle modulated the inverse regulation of topoisomerase IIα and JWA protein expression. NCI-H460 cells were plated on 60 mm plates in DMEM (10% fetal bovine serum), which was changed to starving DMEM (without serum) for 24 h or 30 h. (**a**) Flow cytometric analysed the change of cell cycle. And the protein levels of JWA and topoisomerase IIα were examined by Western blotting. (**c**) and (**d**) Then NCI-H460 cells were treated with 200 nM nocodazole, respectively for 12 h, 18 h and 24 h. (**b**) Flow cytometric analysed the cell cycle. (**e**) and (**f**)The expression of JWA and topoisomerase IIα were examined by Western blotting. β-actin expression served as a loading control. Error bars represent the mean ± SD of triplicate experiments. Statistical differences to the controls were shown as *p < 0.05, **p < 0.01 and ***p < 0.001.

Considering both JWA and topoisomerase IIα are related to tumor cell proliferation and division, we speculated that their mutual interaction might be regulated by cell cycle progression. By using serum starving to arrest cells to G0 phase (Fig. 5a), the expression of JWA was down-regulated after 24 h and then reversed 6 h later. The change of topoisomerase IIα protein was as the same as JWA (Fig. 5c and d). Nocodazole, a mitotic inhibitor, has the effect on majority of NCI-H460 cell synchronization in G2/M phase (Fig. 5b)^30^. In our study, the protein levels of JWA were decreased while increased of topoisomerase IIα when treated with 200 nM of nocodazole for indicated time (Fig. 5e,f).

### Synergistic effect of JWA and topoisomerase IIα on suppression of lung cancer cell migration and invasion

**Figure 6:**
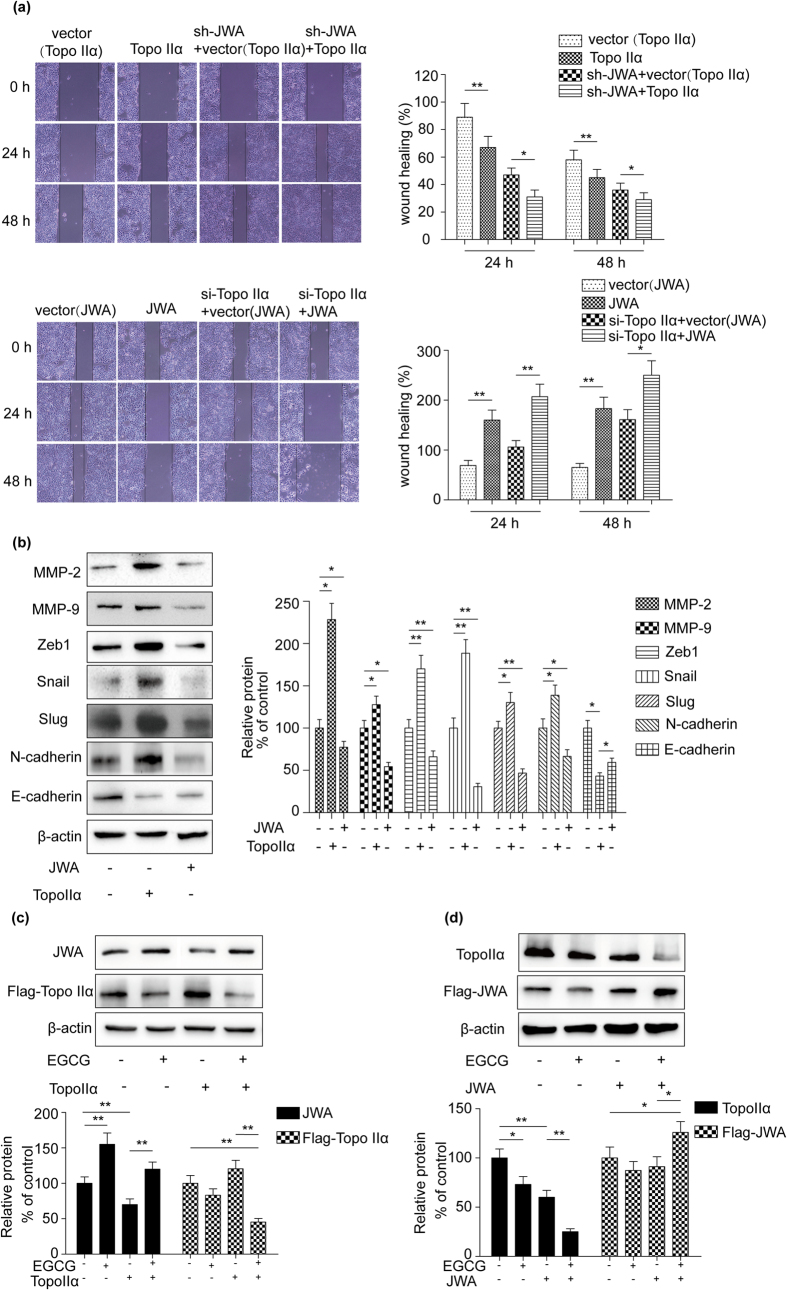
EGCG regulated the interaction between JWA and topoisomerase IIα and their synergistic effect on inhibition of NCI-H460 cells migration and invasion. (**a**) NCI-H460 cells were transfected with siRNA-topoisomerase IIα (100 pmol), shRNA-JWA, JWA and topoisomerase IIα plasmids (4 μg) as well as the control vector. Migration ability of the cells at various time points after transfection (24 h, 48 h) was assessed by scratch migration assay. (**b**) The JWA or topoisomerase IIα plasmid (4 μg) was transiently transfected into NCI-H460 cells. 24 hours later, target proteins in cell lysates were detected by immunoblotting using antibodies against MMP-2/9, N- cadherin, ZEB1, slug, snail and E-cadherin. β-actin expression served as a loading control. (**c**) and (**d**) NCI-H460 cells were transfected with Flag-JWA , Flag- topoisomerase IIα and Flag-vector plasmids, (4 μg) in the presence or absence of EGCG (40 μM) for 24 h. The cells were harvested and lysed for the detection expression of JWA or topoisomerase by Western blot analysis. β-actin expression served as a loading control. Error bars represent the mean ± SD of triplicate experiments. Statistical differences to the controls were shown as *p < 0.05, **p < 0.01.

As described above, topoisomerase IIα is overexpressed in many types of cancer, its down-regulation is a therapeutic mechanism of cancer. Conversely, JWA is usually served as a tumor suppressor and its level is relatively lower in many types of tumor. To study the potential synergistic role of JWA and topoisomerase IIα in lung carcinogenesis, we first investigated the involvement of topoisomerase IIα and JWA in NCI-H460 cells migration using shRNA-JWA, siRNA-topoisomerase IIα, JWA, topoisomerase IIα plasmids and the control vector. A scratch migration assay revealed that topoisomerase IIα deficiency and JWA overexpression cells showed a marked delay in wound closure; Moreover, the capacity of cell migration was increased in sh-JWA transfected cells. Co-transfection of topoisomerase IIα and sh-JWA further accelerated the wound closure (Fig. 6a).

EMT (Epithelial-mesenchymal transition) endows cells with migration and invasion abilities^31^. Considering that downregulation of JWA affected NCI-H460 cells migration, we sought to determine whether JWA affected the expression of EMT signatures. We observed that the expression of MMP-2/9, N-cadherin, ZEB1, slug and snail was down-regulated while E-cadherin was up-regulated after transfection of JWA to NCI-H460 cells. As expected, when cells overexpressed topoisomerase IIα, the levels of MMP-2/9, N- cadherin, ZEB1, slug and snail was higher than that of control, but E-cadherin was at the lower level (Fig. 6b). Taken together, JWA and topoisomerase IIα had a synergistic effect on inhibition of lung cancer cell migration and invasion.

### EGCG regulated the interaction between JWA and topoisomerase IIα

To confirm whether EGCG participated in regulating the cross-talk between JWA and topoisomerase IIα in NCI-H460 cells, cells were transfected with JWA or topoisomerase IIα plasmids, in the presence or absence of EGCG treatment. When cells were transfected with topoisomerase IIα in the presence of EGCG, the down-regulated JWA level was reversed (Fig. 6c). Meanwhile, EGCG was able to further decrease topoisomerase IIα protein expression induced by JWA (Fig. 6d). These results indicated that EGCG was involved in regulating the interplay between JWA and topoisomerase IIα in NCI-H460 cells.

## Discussion

As a powerful cancer chemopreventive agent, EGCG can regulate series of molecular. Topoisomerase IIα is one of those targets. Previous studies showed that EGCG is a topoisomerase inhibitor and poison to topoisomerase II^25^,^26^,^27^. In the present study, we found that EGCG could suppress topoisomerase IIα expression in the post-transcriptional level in NSCLC *in vitro* and *in vivo*. We also found that EGCG could induce JWA expression. JWA is a potential tumor suppressor. The inverse regulation of topoisomerase IIα and JWA by EGCG may add a novel chemopreventive property to EGCG. It is also interesting to find that JWA and topoisomerase IIα are reciprocally down-regulated in several tumour cell lines including NSCLC cells. To our knowledge, this is the first report uncovering the converse-correlation between JWA and topoisomerase IIα, and the potential mechanisms involved.

There are multiple cis-action elements, such as stress response element (SRE) and heat shock response element (hRE) existed within the identified promoter sequence of JWA^8^,^10^. JWA expression is dependent upon generation of intracellular ROS and protects cells against ROS-associated DNA damage^10^,^11^,^12^. EGCG can be a powerful antioxidant to affect intracellular ROS level. The induction of JWA by EGCG could be due to its antioxidant property. Previous research found that EGCG could affect topoisomerase enzyme in a redox-dependent way^26^. Our unpublished data also showed that antioxidants NAC and H_2_O_2_ affected JWA and topoisomerase IIα expression. These data indicated that redox ingredients, such as ROS, might regulate the expression of JWA and topoisomerase IIα.

We observed that JWA and topoisomerase IIα were mutually down-regulated in various cancer cell lines including NSCLC cells and human NSCLC specimens (Fig. 2 and Supplement Figure S1). Overexpression of topoisomerase IIα did not affect the stability of JWA in the cells incubated with MG-132 or leupeptin. Topoisomerase IIα regulated JWA expression at the transcriptional level. Conversely, JWA-mediated topoisomerase IIα degradation was also modulated in a post-translation level and via proteasome and lysosome pathways. The activity of topoisomerase IIα is regulated by posttranslational modification or by interaction with regulatory proteins^32^. Topoisomerase contains a less conserved and largely disordered C-terminal domain (CTD). It is endowed with a main function associated with posttranslational modification and by interaction with regulatory proteins which may result in changes in the half-life of the protein^33^. Considering these data, it is worthy studying whether JWA influenced the CTD of topoisomerase IIα in the future. Besides, JWA is translocated into the nucleus by the carrier protein XRCC1 and protects XRCC1 protein from ubiquitination by proteasome^12^. Nevertheless, little research has been conducted concerning the degradation mechanisms of JWA.

The full length cDNA sequence of JWA contains 2114 nucleosides and a 567 base pairs of open reading frame which encoding 188 amino acids. In JWA protein structure, there are three transmembrane domains (TMD) which include 23 amino acids in each domain (36–58, 64–86, 108–130). Two protein kinase C (PKC) phosphorylational sites (SDR-SLR) are found at the both side of TMD. Beside the first TMD, the other two TMDs possess secondary structures such as random coils and extend strands. Based on JWA structural domain, we built up JWA protein with four fragments (1–60, 1–90, 1–140, 141–188) for identification of binding sites between JWA and topoisomerase IIα. We found that the 90–188 amino acid of JWA played a major function for topoisomerase IIα degradation. It was noteworthy that the third TMD (108–130) was included in 90–188 amino acids. The effect of the TMD of JWA on topoisomerase IIα degradation deserves to be investigated. Future studies will also be needed to determine which, if any, of sites are necessary for the interaction between JWA and topoisomerase IIα.

The conversed correlation of JWA and topoisomerase IIα led us to explore the direct interaction between them. Usually, topoisomerase IIα is located in nucleus while JWA largely presented in cytoplasm^12^,^34^. As Supplement Fig. 2 showed, there was no overlap of JWA localization with topoisomerase IIα. It also failed to detect complex formation between JWA and topoisomerase by immunoprecipitation (Supplement Figure S3). These results indicated that there was no direct interaction between JWA and topoisomerase IIα. It led us speculate whether cell cycle affect their regulation. Topoisomerase IIα is a critical regulator of G2/M transition and highly expressed at this phase. Defects in topoisomerase IIα can lead to a delay in cell cycle progression^19^,^34^. As described above, JWA protein is a novel microtubule-associated protein. The building block of a microtubule is the tubulin subunit that is a heterodimer of α- and β-tubulin. JWA binds to β-tubulin strongly and has completely parallel intracellular distributions^35^. The preliminary data also show that JWA is associated structurally with cell shape, movement and mitosis. It is reasonable that JWA may exert similar functions as microtubule or as a partner of microtubule. In the current study, when cells were arrested in G2/M, JWA and topoisomerase IIα showed an opposite trend in protein expression. However, they had the synchronous expression pattern in serum-starved cells arrested in G0. This indicated that the converse regulation of JWA and topoisomerase IIα was occurred in G2/M which might be controlled by mitosis process. Detailed evidence needs to be provided by further investigation.

An increasing body of evidence demonstrates that down-regulation of JWA increases malignant phenotypes in a variety of cancers such as sarcomas and cervical carcinoma^9^,^36^. Cancer cells with EMT signatures, as evidenced by the decrease of cell adhesion molecules and overexpression of EMT inducers, display increased capability of metastasis^37^,^38^. In hepatocellular carcinoma cells, JWA down-regulation enhances cancer progression through the induction of MMP-2 activity and elevated FAK expression^13^. Also, JWA suppresses tumor angiogenesis via Sp1-activated MMP-2 in human gastric cancer^39^. The data presented here, indicated that knockdown of topoisomerase IIα and overexpression of JWA suppressed lung cancer cell migration and invasion abilities, which might be partially ascribed to elevated E-cadherin expression and decreased MMP-2/9, N- cadherin, ZEB1, slug and snail levels.

In summary, this work uncovers a possible mechanism for the inverse correlation between JWA and topoisomerase IIα, in which it could be regulated by EGCG in NSCLC. Although a definite co-action of these two proteins is yet to be proven clearly, these findings may provide potential chemoprevention targets for NSCLC. Furthermore, it also leads to a new strategy of EGCG in lung cancer prevention.

## Methods

### Cell culture and reagents

NSCLC A549 and NCI-H460 cells were purchased from the Chinese Academy of Sciences Committee on Type Culture Collection Cell Bank (Shanghai, China) .Cell lines have been tested and authenticated by the centre. These cells were cultured in Dulbecco’s Modified Eagle Medium (DMEM, GIBCO, USA) supplemented with 10% fetal bovine serum (Gibco, Grand Island, NY) and 1% penicillin/streptomycin (Beyotime Institute of Biotechnology, Shanghai, China). Cells were maintained at 37 °C in a humidified 5% CO_2_ atmosphere. EGCG were purchased from Sigma-Aldrich (St. Louis, MO, USA). Nocodazole, MG132 and Leupeptin were obtained from Beyotime Institute of Biotechnology (Shanghai, China).

### Plasmids and transient transfection

pIRES-Flag-topoisomerase IIα plasmid was provided by Dr. Lou (Mayo Clinic, MN, USA). The FLAG-JWA 1 (1–60), FLAG –JWA 2 (1–90), FLAG –JWA 3 (1–140), FLAG-JWA 4 (141–188), Flag-JWA, FLAG-vector, shJWA and shJWA vectors were provided by Dr. Jianwei Zhou (Nanjing Medical University, Nanjing, China). Transient transfection was carried out with Lipofectamine 2000 reagent (Invitrogen, Carlsbad, Calif, USA). All steps were performed following the manufacturer’s instructions.

Cells (2 × 10^5^) were transfected with predesigned human topoisomerase IIα siRNA or siRNA control (100 pM) (RiboBio, Guangzhou, China) in 35-mm culture plates using Lipofectamine 2000 reagent (Invitrogen, Carlsbad, Calif, USA).

### Tissue specimens

Institutional approval was obtained from the review boards of Nanjing Medical University prior to this study. NSCLC tissues and matched non-cancerous tissues were obtained from postoperative patients in the Nanjing First Hospital. Tissues were grinded in liquid nitrogen and then extracted protein or RNA for analysis. Written informed consent was obtained from each patient prior to tissue acquisition. Institutional approval was obtained from the Ethical Review Board of Nanjing Medical University (Nanjing, China) prior to this study. Experiment was carried out in accordance with the approved guidelines.

### Serum starvation assay

NCI-H460 cells were cultured in normal medium (DMEM with 10% fetal bovine serum). When cells were 70% confluent, they were then serum-starved for the indicated time (DMEM without serum). Protein was harvested and used for Western blot analysis.

### Western blot analysis

Total cell lysates were prepared with a lysis buffer including 1 mM DTT, 0.1% protease inhibitor and 5 mM PMSF (KeyGEN, Nanjing, China). Protein concentration was checked by BCA Protein Assay Kit (Beyotime, China). The precipitated proteins in the cell lysates were separated by sodium dodecyl sulfate-polyacrylamide gel electrophoresis (SDS-PAGE) gel and transferred to polyvinylidene difluoride membranes (Millipore, Billerica, USA). The primary antibodies were: rabbit polyclonal anti-topoisomerase IIα (Abcam, Cambridge, Britain), anti-anti-N-cadherin (Santa Cruz Biotechnology, USA), anti-snail (Affinity Biosciences, USA), anti-slug (Affinity Biosciences, USA), anti-ZEB1 (Affinity Biosciences, USA), anti-E-cadherin (Santa Cruz Biotechnology, USA), anti-MMP2/9 (Abcam, Cambridge, Britain), β-actin (BOSTER, Wuhan, China), monoclonal anti-flag (sigma, St. Louis, USA), anti-anti-JWA was kindly provided by Dr. Jianwei Zhou (Nanjing Medical University, Nanjing, China). Horseradish peroxidase-conjugated affiniPure goat anti-rabbit or anti-mouse IgG (ZSGB-BIO, China) was the second antibody. Immunoreactive proteins were visualized using the Chemiluminesence Western blotting detection reagents (Cell Signaling Technology, Danvers, MA, USA).

### Real-Time Polymerase Chain Reaction

Total RNA was extracted using the TRIzol reagent (Invitrogen, Carlsbad, CA, USA) following the manufacturer’s protocol. RNA (500 ng) was used for the reverse transcription reaction using the PrimeScriptTM RT Master Mix (TaKaRa Bio Technology). Real-time PCR was performed using SYBRR Premix Ex TaqTMII (TaKaRa BioTechnology) on Applied Biosystems 7300 Real Time PCR System (Applied Biosystems, Foster City, CA) according to manufacturer’s protocol. Expression of mRNA was calculated using the 2−△△Ct method and normalization with GAPDH. Primer sequences are listed below: 5’- CAAGGTCATCCATGACAACTTTG-3’ (forward) and 5’- GTCCACCACCCTGTTGCTGTAG -3’ (reverse) for GAPDH; 5’-TCATGTTGGCGAGCTATTTCC -3’ (forward) and 5’- AGGTTCCGAAGTCTCAACGAT -3’ (reverse) for JWA; 5’- ggcccaagtccagttaaaca -3’ (forward) and 5’-CAGAAAACGATGTCGCAGAA -3’ (reverse) for topoisomerase IIα.

### Scratch migration assay

H460 cells were transfected in 6-well plate. After transfection for 48 h (time 0), the cells were scraped with a sterile 10 μl pipette tip across each well and washed with phosphate buffered saline to remove loose cells. Cell migration into the empty space was followed after 24 h and 48 h and photographed. The wound closure was calculated by the average distance between the two wound edges.

### Tumor xenograft model

A total of 16 (3–5wk old) female BALB/c nude mice were obtained from Slac Laboratory Animal (Shanghai, China). A549 cells (5 × 10^6^) were harvested and injected into the dorsal of the mice. The tumor size was checked twice per week. Tumor volume was calculated using the following formula: volume (mm^3^) = length × width × width/2. When the average volume reached 100 mm^3^, the mice were randomly divided into 2 groups (8 mice/group) as follows: control (normal saline, 0.1 ml/10 g), EGCG (20 mg/kg). Agents were administered twice a week for 4 weeks through intraperitoneal injection. All animals handled in strict accordance with procedures approved by The Center for Hygienic Analysis and Detection of Nanjing Medical University, and the Ethical Review Board was acquired prior to this study.

### Statistical analysis

Data were presented as the mean ± standard deviation (SD) of three independent experiments. Comparisons between quantitative variables were performed using a student’s two-tailed non-paired t-test or one-way analysis of variance (ANOVA). Statistical significance was set at *P < 0.05, **P < 0.01 and ***P < 0.001. Statistical analysis was carried out using the SPSS 17.0 software.

## Additional Information

**How to cite this article**: Li, Y. *et al*. EGCG regulates the cross-talk between JWA and topoisomerase IIα in non-small-cell lung cancer (NSCLC) cells. *Sci. Rep*. **5**, 11009; doi: 10.1038/srep11009 (2015).

## Supplementary Material

Supplementary Information
