# Correction: EGCG regulates the cross-talk between JWA and topoisomerase IIα in non-small-cell lung cancer (NSCLC) cells

**DOI:** 10.1038/s41598-026-58775-6

**Published:** 2026-07-06

**Authors:** Yuan Li, Xin Shen, Xueming Wang, Aiping Li, Pengqi Wang, Pan Jiang, Jianwei Zhou, Qing Feng

**Affiliations:** 1 Department of Nutrition and Food Hygiene, School of Public Health, Nanjing Medical University, Nanjing, Jiangsu 211166 China; 2 Rizhao Centers For Disease Control and Prevention, Rizhao, Shandong 276826 China; 3 Department of Molecular Cell Biology and Toxicology, Cancer Center, School of Public Health, Nanjing Medical University, Nanjing, Jiangsu 211166 China

Correction to: *Scientific Reports* 10.1038/srep11009, published online 05 June 2015

This Article contains errors.

In the Original Article, Figure 6a is erroneously shown for the 0 h panel of sh-JWA + Topo IIα group and the 24 h panel of si-Topo IIα + JWA group. This mistake was an inadvertent oversight arising from bulk figure replacement and processing during manuscript revision. In the Original Article Figure 6. EGCG regulated the interaction between JWA and topoisomerase IIα and their synergistic effect on inhibition of NCI-H460 cells migration and invasion. (a) NCI-H460 cells were transfected with siRNA-topoisomerase IIα (100 pmol), shRNA-JWA, JWA and topoisomerase IIα plasmids (4 μg) as well as the control vector. Migration ability of the cells at various time points after transfection (24 h, 48 h) was assessed by scratch migration assay. The correct Figure 6a is shown below:

**Fig. 6:**
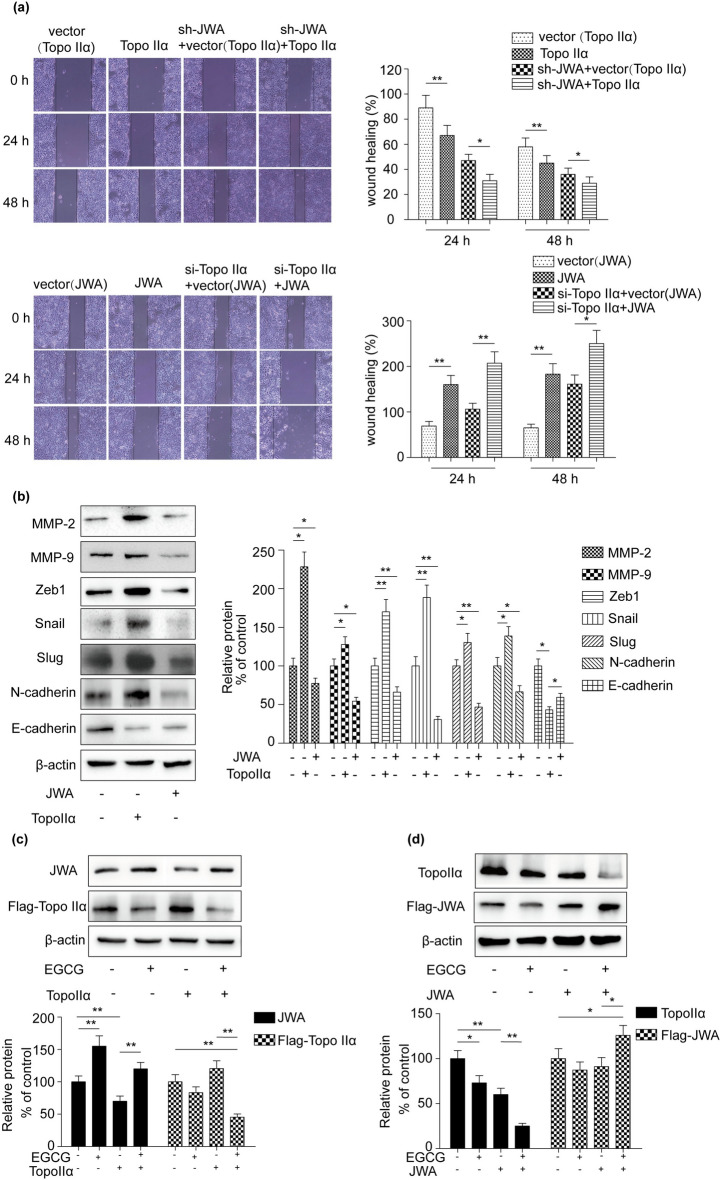
EGCG regulated the interaction between JWA and topoisomerase IIα and their synergistic effect on inhibition of NCI-H460 cells migration and invasion. (**a**) NCI-H460 cells were transfected with siRNA-topoisomerase IIα (100 pmol), shRNA-JWA, JWA and topoisomerase IIα plasmids (4 μg) as well as the control vector. Migration ability of the cells at various time points after transfection (24 h, 48 h) was assessed by scratch migration assay. (**b**) The JWA or topoisomerase IIα plasmid (4 μg) was transiently transfected into NCI-H460 cells. 24 h later, target proteins in cell lysates were detected by immunoblotting using antibodies against MMP-2/9, N- cadherin, ZEB1, slug, snail and E-cadherin. β-actin expression served as a loading control. (**c**) and (**d**) NCI-H460 cells were transfected with Flag-JWA, Flag-topoisomerase IIα and Flag-vector plasmids, (4 μg) in the presence or absence of EGCG (40 μM) for 24 h. The cells were harvested and lysed for the detection expression of JWA or topoisomerase by Western blot analysis. β-actin expression served as a loading control. Error bars represent the mean ± SD of triplicate experiments. Statistical differences to the controls were shown as *p < 0.05, **p < 0.01.

The conclusions of the Original Article are not affected. The corrected panels confirm the same overall results as originally reported.

