# Supplementary material for: EGCG regulates the cross-talk between JWA and topoisomerase IIα in non-small-cell lung cancer (NSCLC) cells
Source: Sci Rep. 2015 Jun 5;5:11009. doi: 10.1038/srep11009 (PMC4457146; doi:10.1038/srep11009)
Supplement: Supplementary Information [file srep11009-s1.doc]

**EGCG regulates the cross-talk between JWA and topoisomerase IIα in non-small-cell lung cancer (NSCLC) cells**

Yuan Li^1^, Xin Shen^1^, ^2^, Xueming Wang^1^, Aiping Li^3^, Pengqi Wang^1^, Pan Jiang^1^, Jianwei Zhou^3^, Qing Feng^1^,*

1. Department of Nutrition and Food Hygiene, School of Public Health, Nanjing Medical University, Nanjing, Jiangsu, 211166, China

2. Rizhao Centers For Disease Control and Prevention, Rizhao, Shandong, 276826，China

3. Department of Molecular Cell Biology and Toxicology, Cancer Center, School of Public Health, Nanjing Medical University, Nanjing, Jiangsu, 211166, China

* Corresponding author: Qing Feng, Ph.D

School of Public Health, Nanjing Medical University, 818 Tianyuan East Rd Nanjing, 211166, Jiangsu, China. Email: [qingfeng@njmu.edu.cn](mailto:qingfeng@njmu.edu.cn); Phone: 86-25-86868455; Fax: 82-25-86868499

**Supplement data**

**Methods**

**Cell culture****.** Human ovarian cancer A2780 cells, gastric cancer BGC823 cells and cervical carcinoma HeLa cells were purchased from the Chinese Academy of Sciences Committee on Type Culture Collection Cell Bank (Shanghai, China). Cell lines have been tested and authenticated by the centre above. These cells were cultured in Dulbecco’s Modified Eagle Medium (DMEM, GIBCO, USA) supplemented with 10% fetal bovine serum (Gibco, Grand Island, NY) and1% penicillin/streptomycin (Beyotime Institute of Biotechnology, Shanghai, China). Cells were maintained at 37°C in a humidified 5% CO2 atmosphere.

**Co-immunoprecipitation.** Protein (1 mg) extracted from cells was incubated with anti-topoisomerase IIα or non-immune mouse IgG at 4°C for 2 h. Protein A/G-agarose beads (Santa Cruz Biotechnology) was added to recover protein-antibody complexes overnight at 4 °C. The mixture was centrifuged at 1,000g for 5 minutes, then the immunoprecipitates were washed with lysis buffer RIPA (Beyotime, China) . The complex were denatured and resolved by SDS-PAGE followed by Western blot analysis using indicated antibodies.

**Immunofluorescence staining.** H460 cells transfected with Flag-JWA or Flag- topoisomerase IIα were fixed with 4% paraformaldehyde and rinsed in phosphate buffered saline supplemented with 0.5% Tween-20 (PBST). For observing intracellular localization of JWA and topoisomerase IIα, cells were permeabilized with 0.2% Triton X-100, and blocked with 2%BSA in PBST for 2h at room temperature. Immunofluorescence labeling was performed using either the combinations of JWA antibody and Alexa Fluor 647-conjugated affinipure goat anti-mouse IgG (Jackson Immuno Research, USA) or anti-topoisomerase IIα and DyLight 549-conjugated affinipure goat anti-rabbit IgG (Jackson Immuno Research, USA). Microscopy was examined with a confocal microscope (Zeiss, Jena, Germany).

**Results**

**JWA and topo****isomerase IIα down-regulated each other in several carcinoma cell lines.** (a) A2780, (b) BGC823 and (c) HeLa cells were transfected with JWA and topoisomerase IIα plasmids. The western blot analysis showed that JWA overexpression suppressed topoisomerase IIα levels in a dose dependent manner. Topoisomerase IIα transfection down-regulated JWA protein expression (Supplement Figure S1).

**The negative regulation between JWA and topoisomerase IIα was not accompanied with changes in** **intracellular localization**

Immunofluorescent microscopy demonstrated that the fluorescence intensity within the cytoplasm (JWA) or nucleus (topoisomerase IIα) did not change after co-transfection with JWA and topoisomerase IIα (Supplement Figure S2).

**There was no direct interaction between JWA and topoisomerase IIα.**

We assessed the formation of JWA-topoisomerase IIα complex by co-immunoprecipitation assay. However, it failed to detect complex formation of JWA and topoisomerase IIα (Supplement Figure S3).

**EGCG might regulate JWA and topoisomerase IIα not through cell cycle.**

Flow cytometric analysis showed that NCI-H460 cells were arrested in S phase slightly when they were treated with EGCG (60 µM). (Supplement Figure S4).

**
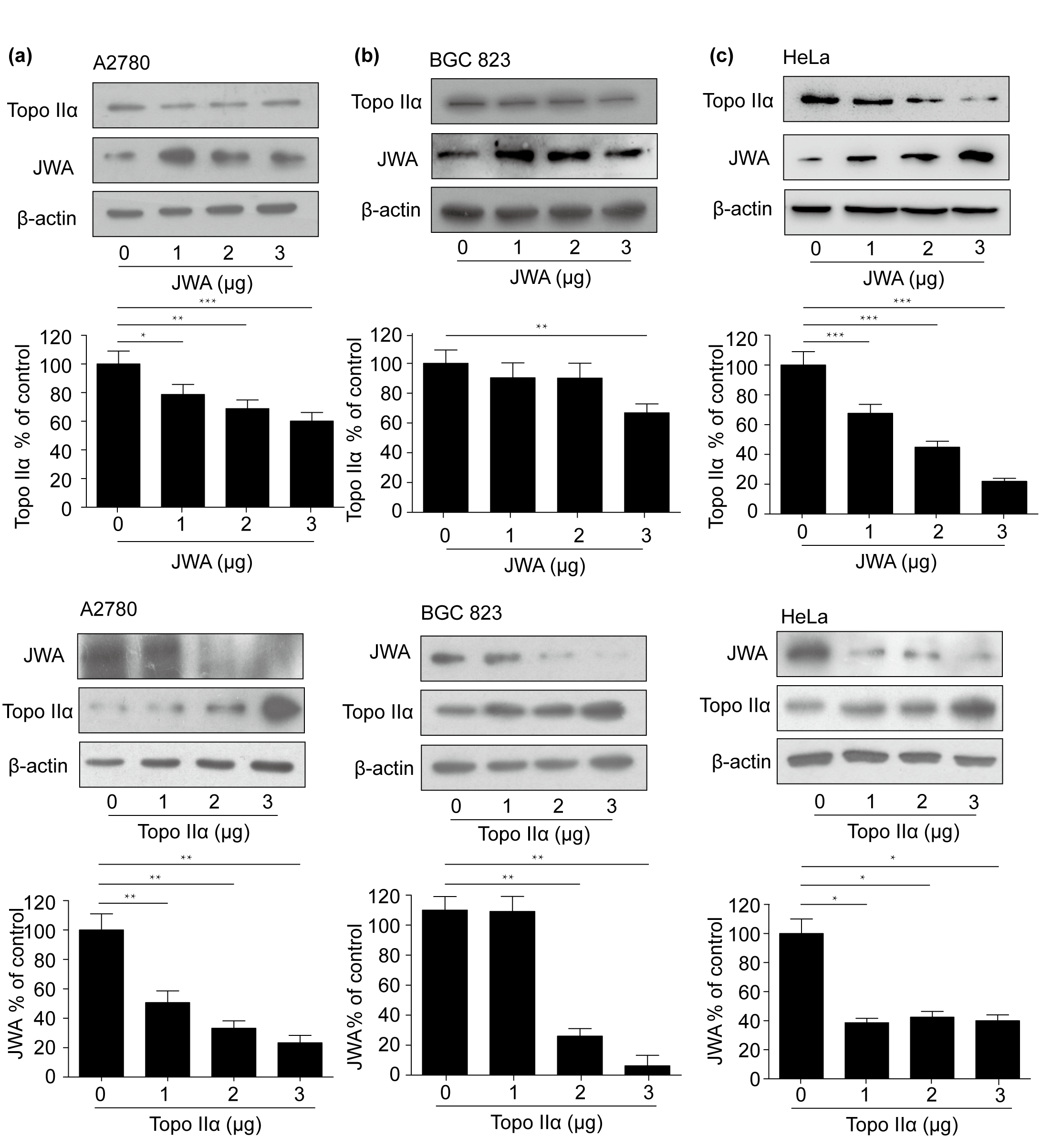
**

**Figure S1** **JWA and** **topoisomerase IIα regulated each other in A2780, BGC823 and HeLa cells.** A2780, BGC823 and HeLa cells were transiently transfected with Flag-JWA or Flag- topoisomerase IIα plasmid (1-4µg). Whole-cell extracts were prepared 24h after transfection and the expression of target proteins was examined. As analyzed by western blot assay, topoisomerase IIα levels were decreased after transfection with JWA plasmid in (a) A2780, (b) BGC823 and (c) HeLa cells. Also, overexpression of topoisomerase IIα does dependently inhibited JWA protein expression in these cells. β-actin was used for the protein loading control. Data were presented as the mean ± SD from three independent experiments. Statistical differences to the controls were shown as *p < 0.05, **p < 0.01, ***p < 0.001.

**
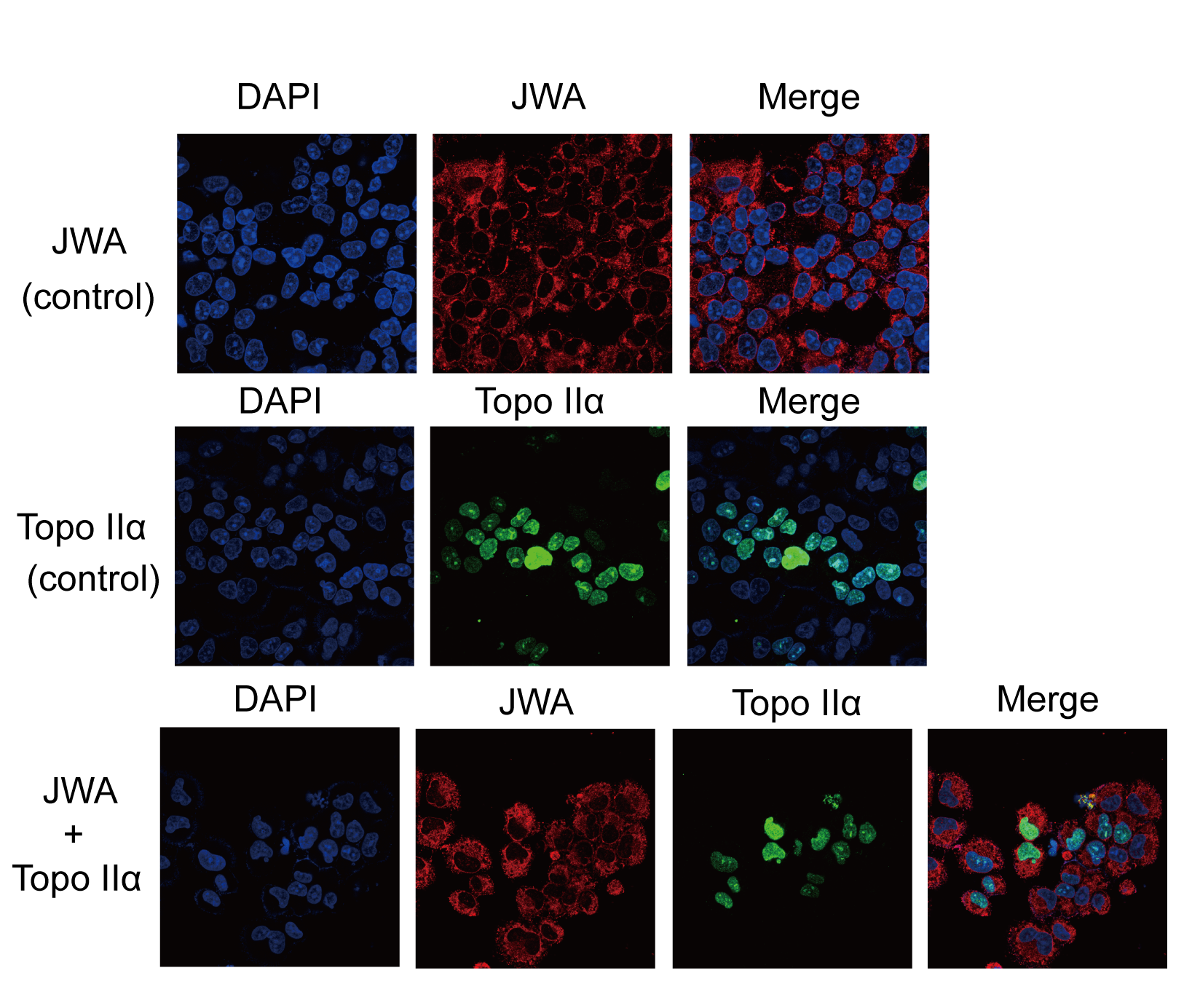
**

**Figure S2 JWA didn’t co-localize with topoisomerase IIα in cells co-transfected with JWA and topoisomerase IIα.** NCI-H460 cells were transfected with JWA and topoisomerase IIα plasmids for 24h. The cells were fixed with 4% paraformaldehyde, permeabilized with 0.2% Triton X-100 and then immunostained with anti-JWA (red) or anti- topoisomerase IIα (green). The nucleus of the cells was indicated by DAPI (blue). The location of JWA and topoisomerase IIα in the cells was observed under a fluorescent microscope.


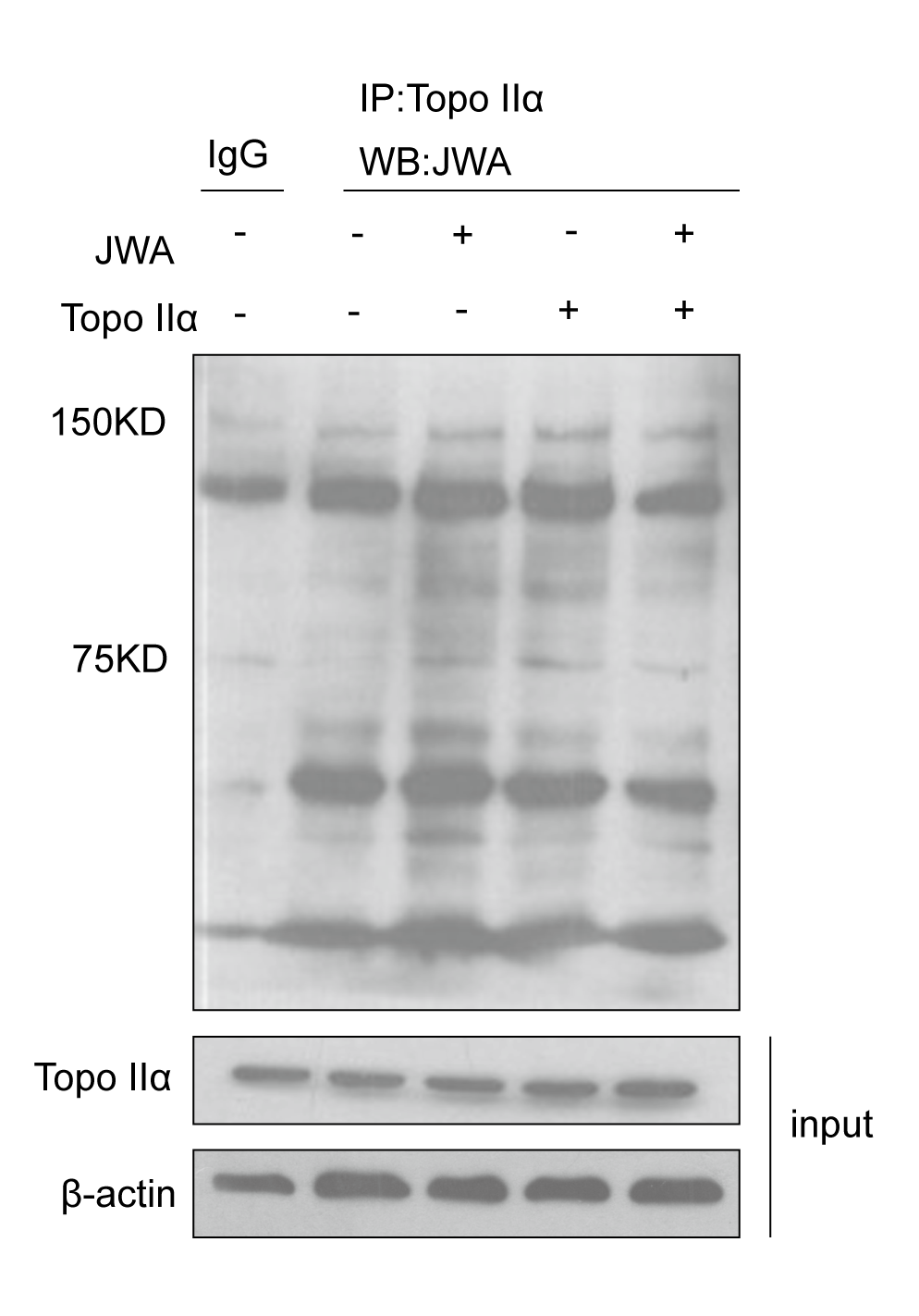


**Figure S3** **JWA interacted with topoisomerase IIα indirectly.** **There was not JWA- topoisomerase IIα complex detected by co-immunoprecipitation analysis.** Flag-JWA and Flag- topoisomerase IIα plasmids were co-transfected into NCI-H460 cells. Lysates were prepared from 24 h post-transfected cells and subjected to IP with anti- topoisomerase IIα antibody followed by IB with indicated antibodies. Densitometry values relative to loading control are given below the blots.


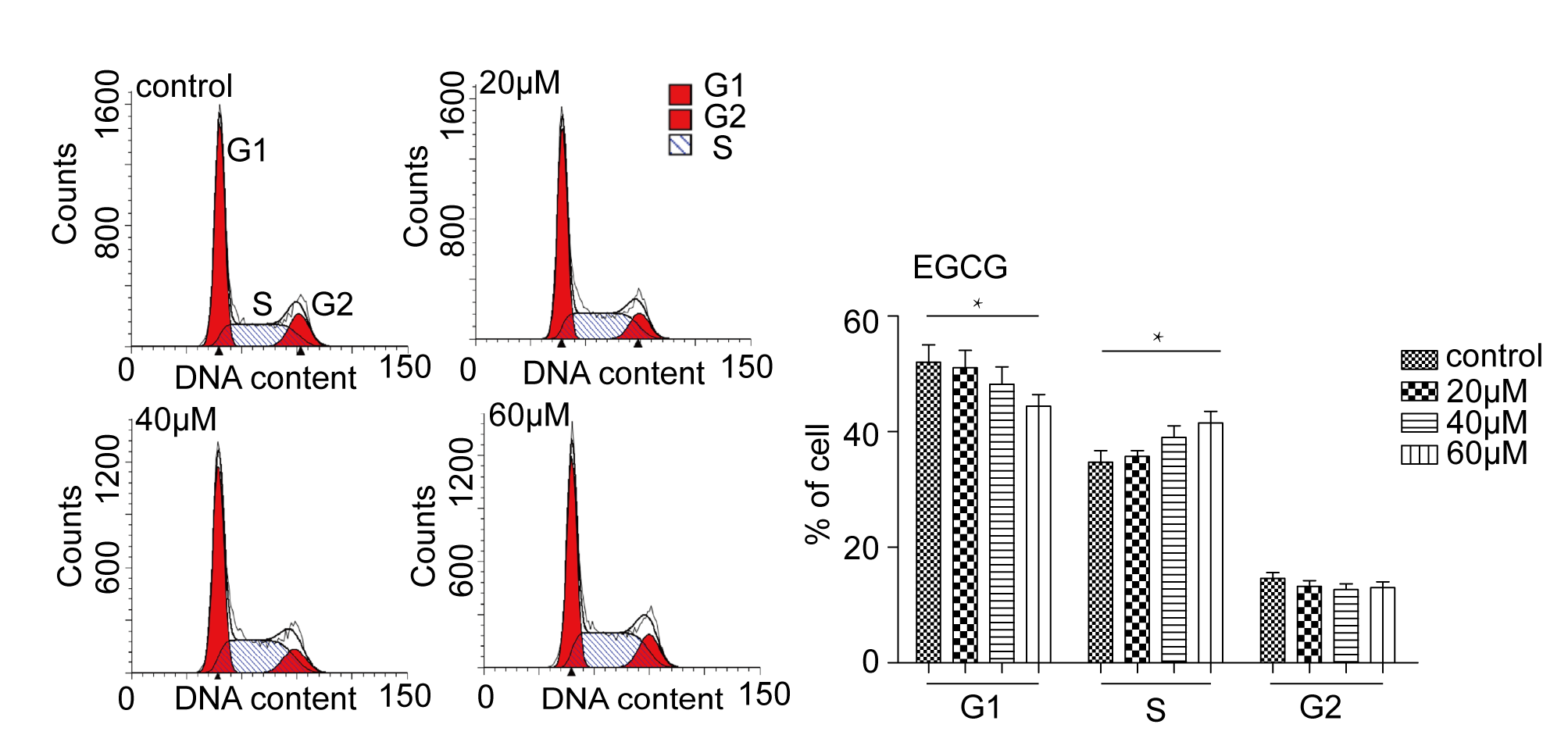


**Figure S4 NCI-H460 cells were arrested in S phase by EGCG.** NCI-H460 cells were treated with EGCG (20-60µM) for 24h. Then flow cytometric analysed the cell cycle.

**Raw cropped gels/blots**

Our results were from several independent experiments (in triplicate for each experiment at least). So we selected the best one for publication. We exhibited part of the raw blots here. These raw data might not be in full accord with the ones for publication (for every bands), however, they certainly showed the same conclusion in the manuscript.

**1. JWA and topoisomerase IIα regulated each other in H460A2780, BGC823 and HeLa cells.**

**
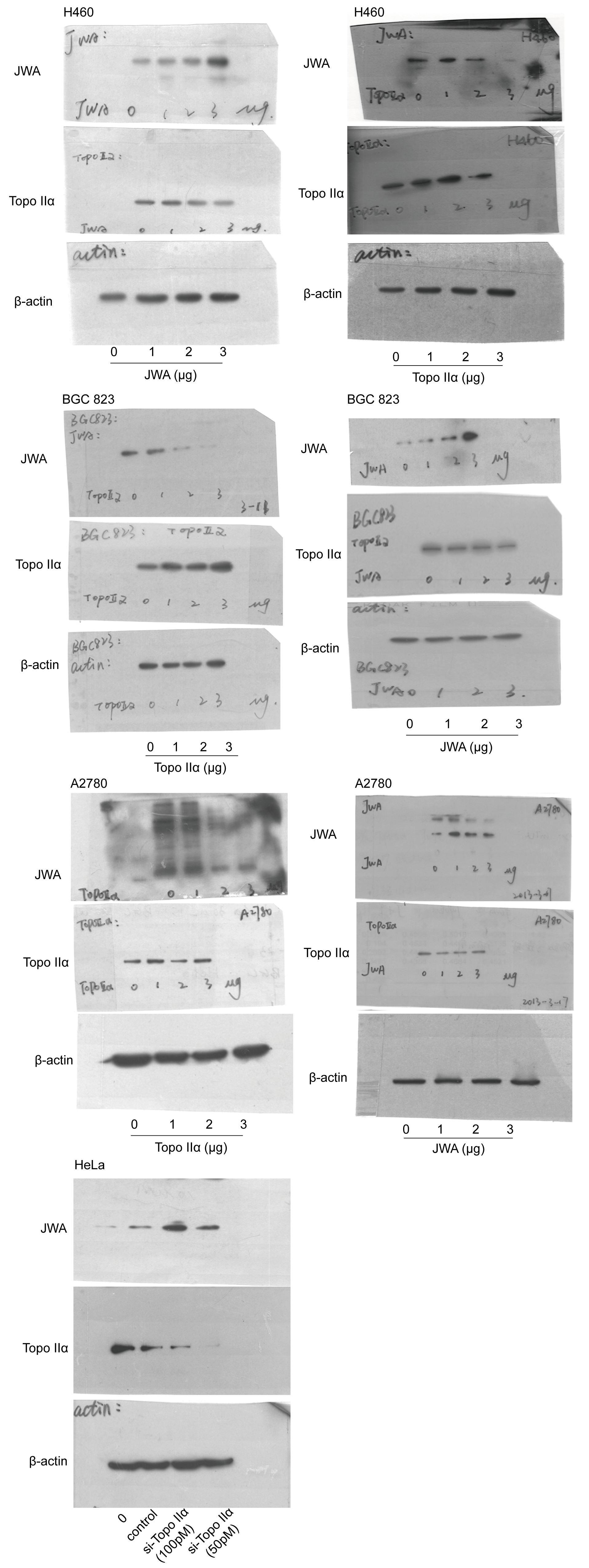
**

**2. EGCG induced JWA protein expression**

**
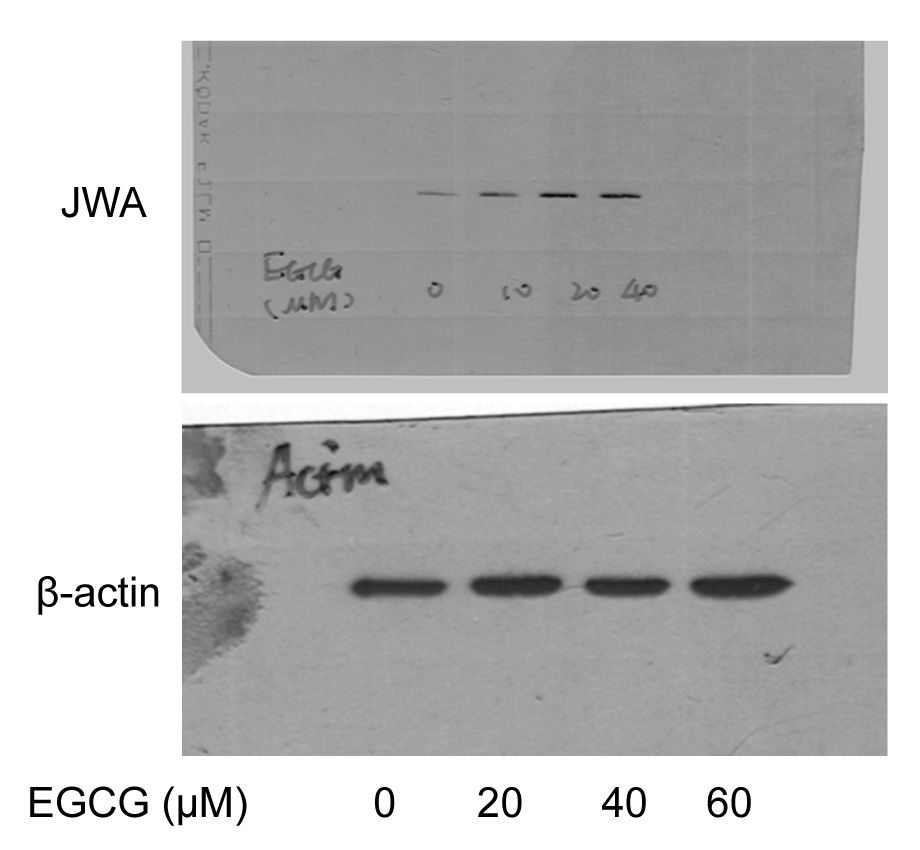
**

**3. Lysomal and proteasomal pathway were involved in JWA-mediated suppression of topoisomerase IIα.**

**
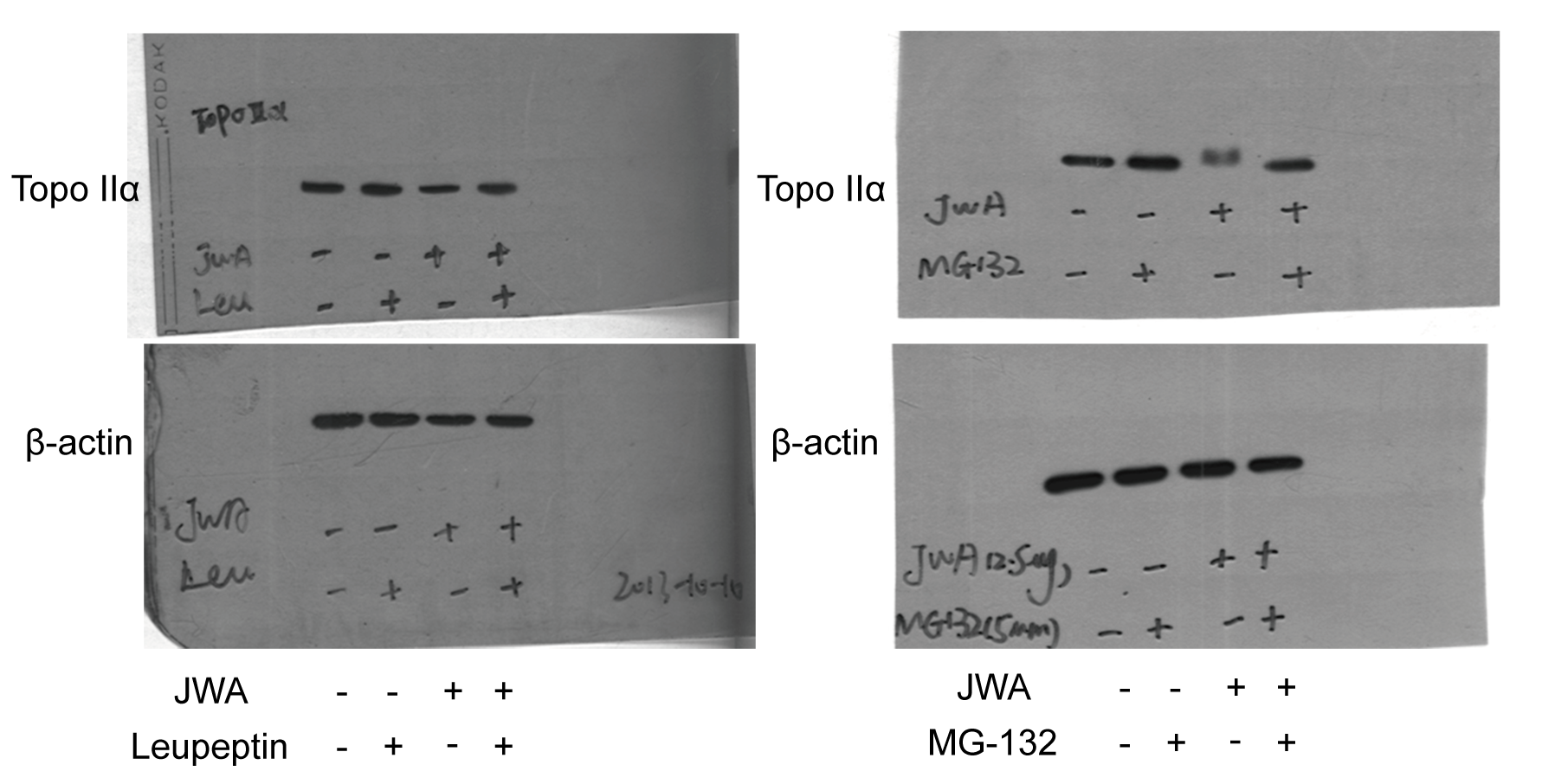
**

**4. The** **amino acid sequence of JWA ranging from 90 to 188 played a crucial role in** **topoisomerase IIα degradation**

**
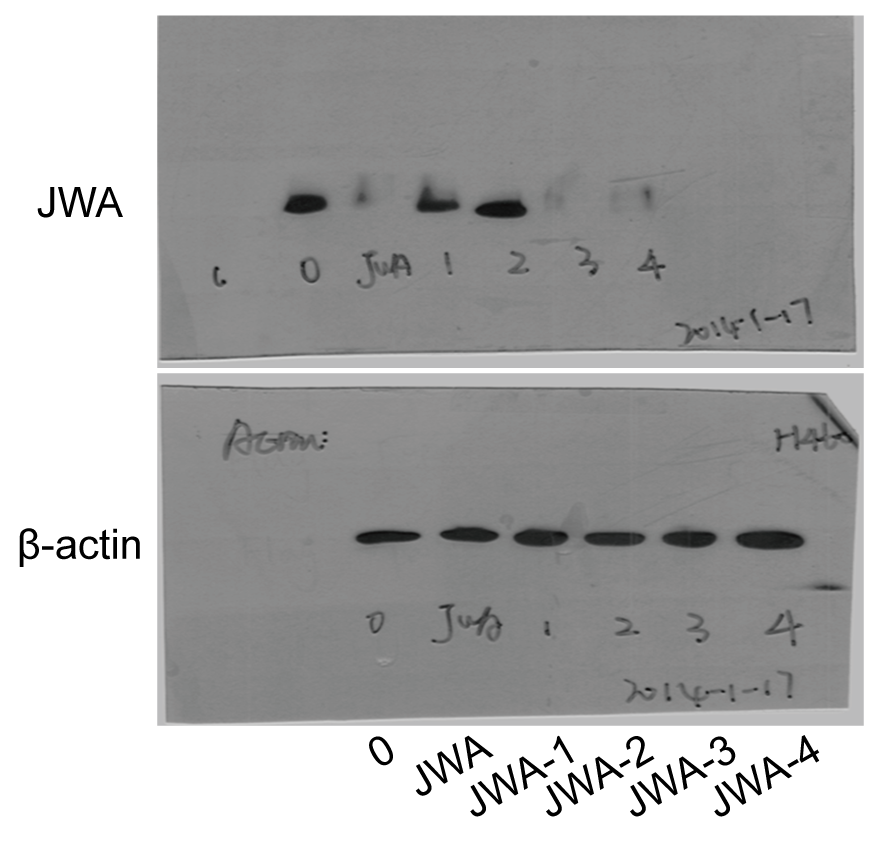
**

**5. The protein level of N-cadherin and transcription factor Snail, Slug and Zeb1 when topoisomerase IIα and JWA are overexpressed.**

**
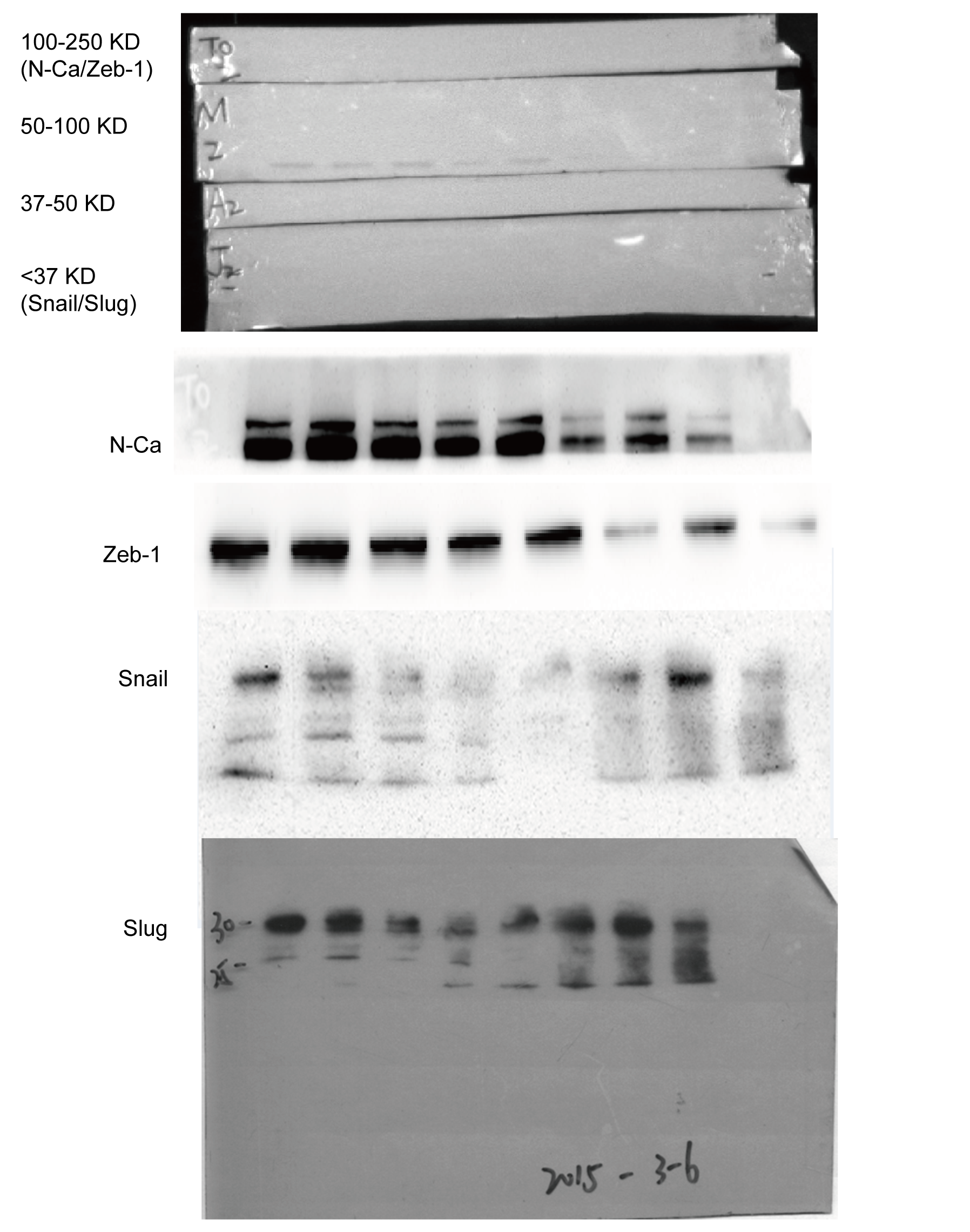
**

**6. EGCG regulated the protein level of topoisomerase IIα and JWA when they are overexpressed.**

**
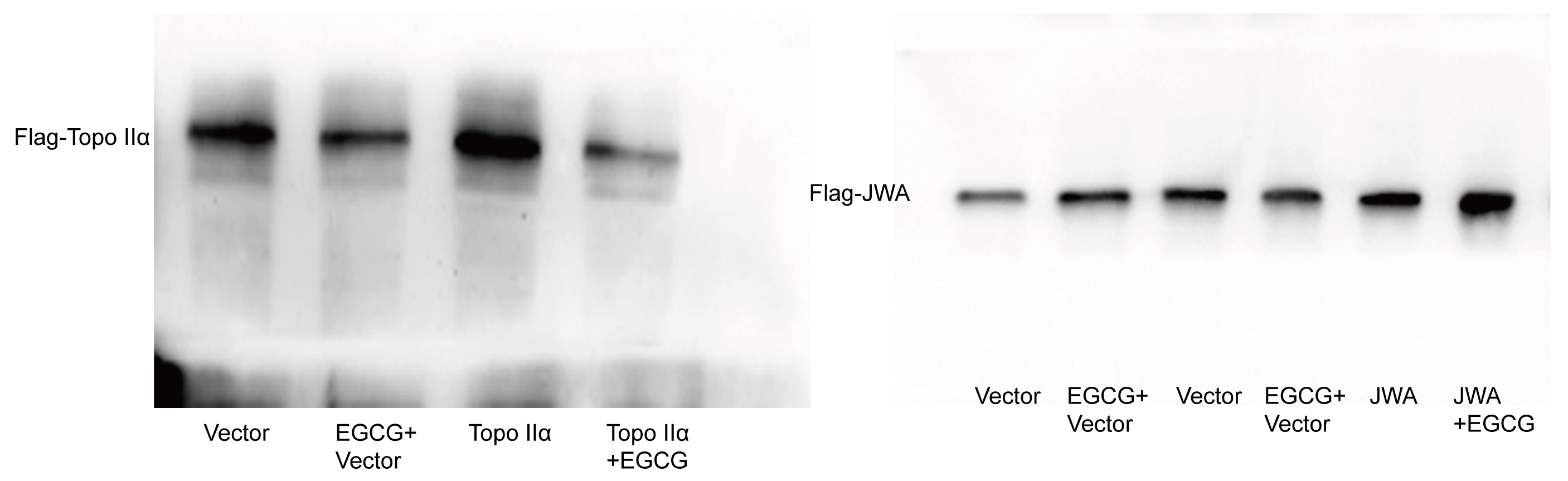
**
